# Treatment of diabetic mice with undenatured whey protein accelerates the wound healing process by enhancing the expression of MIP-1α, MIP-2, KC, CX3CL1 and TGF-β in wounded tissue

**DOI:** 10.1186/1471-2172-13-32

**Published:** 2012-06-18

**Authors:** Gamal Badr, Badr M Badr, Mohamed H Mahmoud, Mohamed Mohany, Danny M Rabah, Olivier Garraud

**Affiliations:** 1 Princes Johara alibrahim center for cancer research, prostate cancer research chair, College of Medicine, King Saud University, Riyadh, Saudi Arabia; 2 Zoology Department, Faculty of Science, Assiut University, 71516, Assiut, Egypt; 3 Vice-Rectorate for Graduate Studies and Research-Visiting Professor Program, King Saud University, Riyadh, Saudi Arabia; 4 Zoology Department, College of Science, King Saud University, Riyadh, Saudi Arabia; 5 Human nutrition Department, National Research Centre, Dokki, Cairo, Egypt; 6 Zoology Department, Faculty of Science, Menoufia University, Menoufia, Egypt; 7 Department of Urology/Surgery, College of Medicine, King Saud University, Riyadh, Saudi Arabia; 8 EA3064—GIMAP, Université de Lyon, F-42023, Saint-Etienne (cedex 2), France

**Keywords:** Cytokines, Diabetes mellitus, Inflammation, Wound healing, Whey protein

## Abstract

**Background:**

Continuous diabetes-associated complications are a major source of immune system exhaustion and an increased incidence of infection. Diabetes can cause poor circulation in the feet, increasing the likelihood of ulcers forming when the skin is damaged and slowing the healing of the ulcers. Whey proteins (WPs) enhance immunity during childhood and have a protective effect on some immune disorders. Therefore, in this study, we investigated the effects of camel WP on the healing and closure of diabetic wounds in a streptozotocin (STZ)-induced type I diabetic mouse model.

**Results:**

Diabetic mice exhibited delayed wound closure characterized by a significant decrease in an anti-inflammatory cytokine (namely, IL-10) and a prolonged elevation of the levels of inflammatory cytokines (TNF-α, IL-1β and IL-6) in wound tissue. Moreover, aberrant expression of chemokines that regulate wound healing (MIP-1α, MIP-2, KC and CX3CL1) and growth factors (TGF-β) were observed in the wound tissue of diabetic mice compared with control nondiabetic mice. Interestingly, compared with untreated diabetic mice, supplementation with WP significantly accelerated the closure of diabetic wounds by limiting inflammatory stimuli via the restoration of normal IL-10, TNF-α, IL-1β and IL-6 levels. Most importantly, the supplementation of diabetic mice with WP significantly modulated the expression of MIP-1α, MIP-2, KC, CX3CL1 and TGF-β in wound tissue compared with untreated diabetic mice.

**Conclusion:**

Our data demonstrate the benefits of WP supplementation for improving the healing and closure of diabetic wounds and restoring the immune response in diabetic mice.

## Background

Type I diabetes (T1D) is a chronic autoimmune disease caused by the specific destruction of pancreatic β-cells, which produce insulin [1], Multiple complications are usually associated with diabetes mellitus [2]. Impaired wound healing represents a severe complication of the disease, which could diminish physical activity and lead to chronic wounds and limb amputation [3]. Previous studies have reported that wound healing involves a complex series of interactions between different cell types, cytokine mediators, and the extracellular matrix. The phases of normal wound healing include hemostasis, inflammation, proliferation, and remodeling [4]. Moreover, many factors that can affect wound healing interfere with one or more phases in this process, thus causing improper or impaired tissue repair. For instance, collagen deposition in acute wounds is impaired in T1D; possibly due to a decreased fibroblast proliferation [5]. The capacity of a wound to heal depends in part on its depth, as well as on the overall health and nutritional status of the individual [6]. Cytokines such as IL-1α, IL-1β, IL-6, and TNF-α are thought to play important roles in wound repair, including the stimulation of keratinocyte and fibroblast proliferation, the synthesis and breakdown of extracellular matrix proteins, fibroblast chemotaxis, and immune response regulation [7]. Diabetic individuals are more susceptible to both wound infection and hyper-inflammation, which cannot be pathogenically separated from the elevated levels of pro-inflammatory cytokines, such as TNF-α and IL-6 [8]. In addition, TNF-α dysregulation in mouse model of diabetic wounds impairs healing, which may involve the enhanced apoptosis and decreased proliferation of fibroblasts [9]. Moreover, overexpression of IL-10, an anti-inflammatory cytokine, decreases the inflammatory response to injury, creating an environment conducive for regenerative adult wound healing [10]. Several chemokines that have been identified as regulators of specific leukocyte accumulation at wound sites [11], such as macrophage inflammatory proteins 1α and 2 (MIP-1α, MIP-2), are major chemoattractants for monocytes/macrophages and play key roles in macrophage infiltration during wound healing [12]. In particular, keratinocyte-derived chemokine (KC) and MIP-2 are believed to participate in the recruitment of neutrophils to sites of inflammation in many tissues [13]. CX3CL1 is expressed as a soluble chemokine and as a membrane-bound form on the surface of inflamed endothelial cells, epithelial cells, macrophages, and vascular smooth muscle cells. The chemokine CX3CL1 and its receptor CX3CR1 are both highly induced at wound sites and mediate skin wound healing by promoting macrophage and fibroblast accumulation and function [14]. Although CX3CL1 directly stimulates angiogenesis, the local microvasculature also depends on several other growth factors, including transforming growth factor-β (TGF-β), which is produced by macrophages in wounds [15] and functions in leukocyte chemotaxis, fibroblast and smooth muscle cell mitogenesis and extracellular matrix deposition during granulation tissue formation [16].

Protein is essential for the maintenance and repair of body tissue. Depleted protein levels cause a decrease in collagen development, slowing the wound healing process. Adequate protein levels help to achieve optimal wound healing rates [17]. Camel whey proteins (WPs) are composed of a heterogeneous group of proteins that include serum albumin, α lactalbumin, immunoglobulin, lactophorin and peptidoglycan recognition protein [18]. Dietary whey supplementation is thought to increase glutathione synthesis and cellular antioxidant defense [19]. Therefore, WP may be a therapeutic tool for oxidative stress-associated diseases [20]. In addition to its immunomodulatory properties and its ability to boost the host defense systems [21], WP fractions are linked to a range of bioactive functions, such as prebiotic effects, the promotion of tissue repair, the maintenance of intestinal integrity, the destruction of pathogens and the elimination of toxins [22]. In addition, a clear modulation of immune functions by several whey protein-derived products has been demonstrated *in vitro* and *in vivo*[23]. Our recent published study revealed that the supplementation of diabetic mice with WP rescued functional, long-lived wound-resident macrophages and improved the healing and closure of diabetic wounds [24]. Additionally, it was found that dietary supplementation with WP enhances the normal inflammatory responses during wound healing in diabetic mice by restoring the levels of oxidative stress and inflammatory cytokines [25]. Recent studies have shown that whey increases antioxidant activity in the body, combats fatigue and inflammation, hastens healing, improves stamina and may discourage related infections because of the immune system-enhancing and natural antibiotic properties of its components [26,27]. Nevertheless, there are few studies that have investigated the influence of WPs in wound healing. The aim of this study was to investigate the potential modulatory effects of the oral administration of WPs on wound healing using a diabetic mouse model.

## Methods

### Preparation of whey proteins

Raw camel milk was collected from healthy female camels (Majaheim) from the Riyadh area in Saudi Arabia. The milk was then centrifuged to remove the cream. The obtained skim milk was acidified to pH 4.3 using 1 N HCl at room temperature and centrifuged at 10,000 x *g* for 10 min to precipitate the casein. The resulting whey, which contained the whey proteins, was saturated with ammonium sulfate to a final saturation of 80% to precipitate the whey proteins. The precipitated whey proteins were dialyzed against 20 volumes of distilled water for 48 hr using a molecular porous membrane with a molecular weight cutoff of 6,000-8,000 kDa. The dialysate containing undenatured whey proteins was lyophilized and refrigerated until use.

### Chemicals

Streptozotocin (STZ) was obtained from Sigma Chemical Co., St. Louis, MO, USA. STZ was dissolved in cold 0.01 M citrate buffer (pH 4.50) and was always freshly prepared for immediate use (within 5 min).

### Animals and experimental design

A total of 60 sexually mature, 12-week-old male Swiss Webster (SW) mice, each weighing 25-30 g, were obtained from the Central Animal House of the Faculty of Pharmacy at King Saud University. All animal procedures were conducted in accordance with the standards set forth in the Guidelines for the Care and Use of Experimental Animals by the Committee for the Purpose of Control and Supervision of Experiments on Animals (CPCSEA). The study protocol was approved by the Animal Ethics Committee of the Zoology Department, College of Science, King Saud University according to the Helsinki principles. All animals were allowed to acclimate to the metal cages inside a well-ventilated room for 2 weeks prior to experimentation. Animals were maintained under standard laboratory conditions (temperature 23 °C, relative humidity 60-70% and a 12-hour light/dark cycle), fed a diet of standard commercial pellets and given water ad libitum. All mice were fasted for 20 hr prior to diabetes induction. Mice (*n* = 40) were rendered diabetic with an intraperitoneal injection (i.p.) of a single dose of STZ (60 mg/kg body weight) in 0.01 M citrate buffer (pH 4.5) [28]. Blood glucose levels were measured 3 consecutive days after STZ injection by cutting off the tip of the tail, squeezing it gently and using OneTouch Ultra (LifeScan, Paris, France). Mice were considered diabetic if glycemia was higher than 220 mg/dl with monitoring initial and final glycemia during wound healing period (Badr, 2012). Mice in the control group (*n* = 20) were injected with the vehicle alone (0.01 M citrate buffer, pH 4.5). The animals were divided into three experimental groups: group 1, control non-diabetic mice that were orally supplemented with distilled water (250 μl/mouse/day for one month by oral gavage); group 2, diabetic mice that were orally supplemented with distilled water (250 μl/mouse/day for one month by oral gavage); and group 3, diabetic mice orally supplemented with undenatured WP (100 mg/kg body weight dissolved in 250 μl/day for one month by oral gavage). Therefore, the supplemented volume for the 3 groups was constant and did not exceed 250 μl per dosage. The optimal dose of WP was determined in our laboratory on the basis of the LD_50_ and several established parameters.

### Excisional wound preparation and macroscopic examination

Following diabetes induction, mice in each group (n = 20) were wounded at the age of 12 weeks. Wounded mice in each group (n = 20) were divided into two subgroups (n = 10 in each subgroup). Ten mice were used for the data presented in this study and the other 10 animals from each group were used for the measurement of hydroxyproline content in the wound sites. After drying for 24 h at 120 °C, the amount of hydroxyproline, a major constituent of collagen in skin wound sites, was measured to index collagen accumulation at the wound site. All the diabetic animals in the diabetic group (n = 20) and in the WP-treated diabetic group (n = 20) were all responded to STZ and diabetes induction was confirmed by monitoring the blood glucose levels throughout the experiment period. Wounding of mice was performed as described previously [29]. Briefly, mice were anesthetized with a single i.p. injection of ketamine (80 mg/kg body weight) and xylazine (10 mg/kg body weight). Blood samples were immediately collected from orbital sinus in a non-heparinized tube and centrifuged for 10 min at 3000 rpm to separate the serum, then stored at 80 °C and later used to biochemical analyses. The hair on the back of each mouse was cut, and the back was subsequently cleansed with 70% ethanol. Six full-thickness wounds (5 mm in diameter, 3-4 mm apart) were made on the back of each mouse by excising the skin and the underlying panniculus carnosus. The wounds were allowed to form a scab.

### Wound closure measurement

Skin biopsy specimens were obtained from the animals at 4, 7, 10, and 13 days post-injury. At each time point, an area that included the scab, the complete epithelial and dermal compartments of the wound margins, the granulation tissue, and parts of the adjacent muscle and subcutaneous fat tissue was excised from each individual wound. Occasionally, a similar amount of skin was taken from the backs of non-wounded normal mice as a control. Each wound site was photographed by single camera digital photogrammetry (SCP) at the indicated time intervals to determine the wound areas. The wound area was determined by wound measurement software (VeV MD, Vista Medical, Winnipeg, Manitoba, Canada). Total surface area yet to be healed was calculated by the principal investigator who was blinded to group assignment. Changes in wound area are expressed as the percentage of the initial wound area. At the indicated time points, tissue from two wounds from each of ten animals (n = 20 wounds) was harvested for RNA analysis, RT-PCR.

### Blood analysis

Blood glucose levels were determined using the AccuTrend sensor (Roche Biochemicals, Mannheim, Germany). Serum insulin levels were analyzed by Luminex (Biotrend, Düsseldorf, Germany) according to the manufacturer’s instructions.

### Biochemical analysis of wound tissue

#### Analysis of wound tissue cytokine levels

Wound tissues from 10 mice/group were pulverized under liquid nitrogen followed by the extraction of protein as described [30]. The levels of IL-1β, IL-6, IL-10 and TNF-α in tissue extracts were measured using commercially available ELISA kits (R&D Systems, Wiesbaden, Germany) according to the manufacturer’s instructions.

### Extraction of total RNA and RT-PCR

Total RNA was isolated from wounded skin samples (10 mice/group) using TRIzol reagent (Invitrogen Life Technologies, France) according to the manufacturer’s instructions. Before reverse transcription, RNA was treated with RNase-free DNase I following the manufacturer’s protocol. cDNA was synthesized from 3 μg of total RNA using a Superscript III RT kit (Invitrogen Life Technologies, France). Unique primer sets for mouse MIP-1α, MIP-2, KC, CX3CL1, TGF-β1 and β-actin were designed (Table 1) based on sequences deposited with the National Center for Biotechnology Information and were synthesized by Invitrogen Life Technologies. PCR was performed using 1 μl of cDNA in a reaction mix with *Taq* polymerase (Invitrogen Life Technologies). PCR was found to be linear between 20 and 35 cycles, and PCR conditions were optimized to allow for a semiquantitative comparison of results. Bands separated on ethidium bromide-stained agarose gels were quantitated from digital images using NIH Image Analysis Software. The intensity of each product was normalized to the intensity of the β-actin product and expressed relative to the levels in injured skin from control non-diabetic mice.

**Table 1:** Sequences of primers used for RT-PCR

**Transcript**	**Sequences**	**Product size (bp)**
MIP-1α	(F) 5’-GCCCTTGCTGTTCTTCTCTGT-3’	258
	(R) 5’-GGCATTCAGTTCCAGGTCAGT-3’	
MIP-2	(F) 5’-GAACAAAGGCAAGGCTAACTGA-3’	204
	(R) 5’-AACATAACAACATCTGGGCAAT-3’	
KC	(F) 5’-GTGTCCCCAAGTAACGGAGA-3’	317
	(R) 5’-TGCACTTCTTTTCGCACAAC-3’	
CX3CLI	(F) 5’GTTGGCTCCTGAGAGTGAGG-3’	301
	(R) 5’-CAAAATGGCACAGACATTGG-3’	
TGF-β1	(F) 5’-CGGGGCGACCTGGGCACCATCCATGAC-3’	405
	(R) 5’-CTGCTCCACCTTGGGCTTGCGACCCA-3’	
β-actin	(F) 5’-TTCTACAATGAGCTGCGTGTGGC-3’	456
	(R) 5’-CTCATAGCTCTTCTCCAGGGAGGA	

*(F)*, Forward primer; *(R)*, reverse primer.

### Statistical analysis

Data were first tested for normality (using Anderson–Darling test) and for variances homogeneity prior to any further statistical analysis. Data were normally distributed and were expressed as the mean ± standard error of the mean (SEM). Significant differences among groups were analyzed using a one- or two-way ANOVA followed by Bonferroni’s multiple comparison tests using PRISM statistical analysis software (GraphPad Software) and data was reanalyzed using a one- or two-way ANOVA followed by Tukey’s post-test using SPSS software, version 17. Differences were considered statistically significant at ^*^P < 0.05, diabetic vs. control; ^+^P < 0.05, diabetic + WP vs. control; ^#^P < 0.05, diabetic + WP vs. diabetic.

## Results

### Administration of camel WP expedites wound closure in diabetic mice

**Figure 1:**
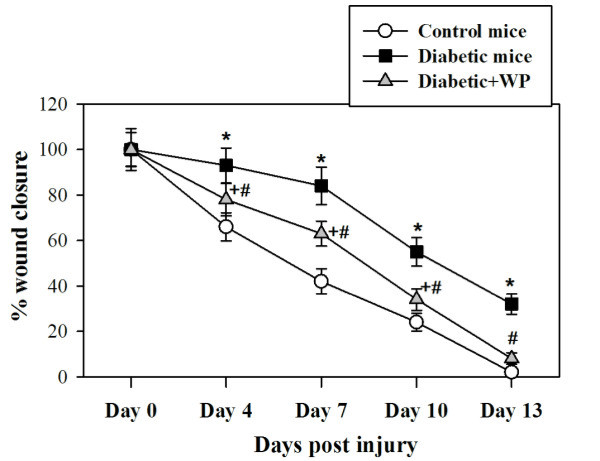
**Macroscopic changes at skin excisional wound sites.** Changes in the percentage of wound closure at each time point compared with the original wound area (day 0) is shown. Accumulated data from 10 individual mice in each group is expressed as the mean percentage of wound closure ± SEM at each time point.

We evaluated the macroscopic changes at skin-excision wound sites in control mice, diabetic mice and diabetic mice supplemented with WP. Pictures were taken on day 0 immediately after the injury. The wound sites exhibited a similar morphology in all 3 experimental groups on day 1 post-injury, and the wounds in the control and diabetic mice supplemented with WP were similarly closed at 13 days post-injury. By contrast, the diabetic mice exhibited delayed wound closure. Changes in the diameters of wounded area throughout the experiment period were monitored in the 3 groups. Accumulated data from 10 individual mice in each group is expressed as the mean percentage of wound closure ± SEM at each time point Figure 1. These results demonstrate that wound closure and healing were accelerated in the diabetic mice supplemented with camel WP compared with untreated diabetic mice, which exhibited delayed wound closure. To optimize the parameters and conditions of the animal models during the experiments, blood glucose and insulin levels in the 3 groups of mice were monitored before and throughout the indicated time points post-injury (Table 2). Glucose levels in the WP-treated diabetic group were significantly lower than in the diabetic group and were higher than in the control group. By contrast, insulin levels were significantly increased in the WP-treated diabetic group compared with diabetic mice throughout the wound healing period.

**Table 2:** Blood glucose and insulin levels

**Blood glucose level (mg/dL)**			
	**Control mice**	**Diabetic mice**	**Diabetic + WP**
Day 0	120 ± 11	360 ± 11.7*	280 ± 11.4 + #
Day 7	148 ± 13.8	370 ± 9.9*	301 ± 10.4 + #
Day 10	129 ± 11.6	345 ± 8.5*	280 ± 11.2 + #
Day 13	151 ± 12.4	377 ± 7.9*	257 ± 9.4 + #
**Blood insulin level (ng/ml)**			
	**Control mice**	**Diabetic mice**	**Diabetic + WP**
Day 0	4 ± 0.35	1.1 ± 0.2	2.4 ± 0.22 + #
Day 7	4.5 ± 0.4	1.9 ± 0.18	2.7 ± 0.19 + #
Day 10	3.9 ± 0.33	2.1 ± 0.2	2.2 ± 0.2+
Day 13	5 ± 0.45	1.5 ± 0.12	3 ± 0.25 + #

*P < 0.05 Diabetic vs control.

‡ P < 0.005 Diabetic + WP vs control.

# P < 0.05 Diabetic + WP vs diabetic.

The levels of blood glucose and insulin were monitored at the indicated time points post-injury. Representative results of 10 individual mice from each group are shown as the mean ± SEM. ^*^P < 0.05, diabetic vs. control; ^+^P < 0.05, diabetic + WP vs. control; ^#^P < 0.05, diabetic + WP vs. diabetic (ANOVA with Tukey’s post-test).

### WP supplementation during diabetes restores the levels of wound tissue cytokines

**Figure 2:**
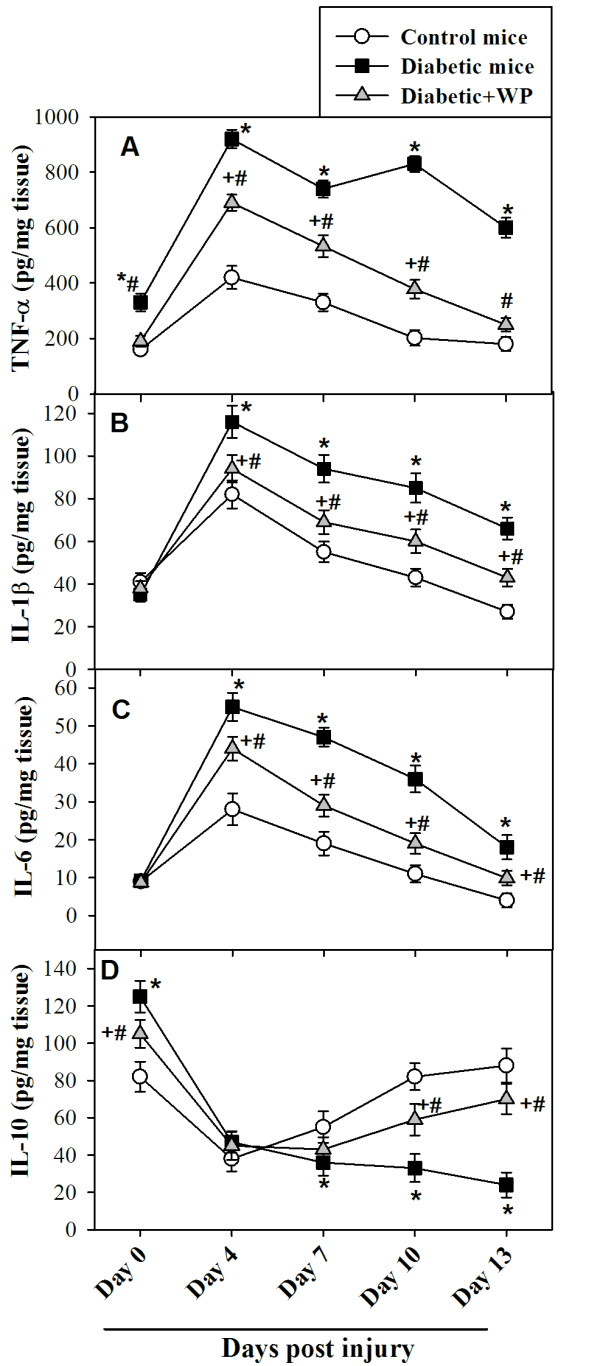
**Profile of pro- and anti-inflammatory cytokines in wound area.** The levels of pro-inflammatory cytokines (TNF-α, IL-1β and IL-6) and an anti-inflammatory cytokine (IL-10) were measured by ELISA in the wound tissues from the 3 groups of mice before wounding (Day 0) and on the indicated days post-wounding. The results are expressed as the mean ± SEM. ^*^P < 0.05, diabetic vs. control; ^+^P < 0.05, diabetic + WP vs. control; ^#^P < 0.05, diabetic + WP vs. diabetic (ANOVA with Tukey’s post-test).

Cytokines are secreted by specific immune cells, carry signals locally between cells and are critical for the wound healing process. Therefore, we monitored the levels of pro- and anti-inflammatory cytokines that control immune cell function during wound healing in the three groups of mice. Data from 10 individual mice from each group are shown in Figure 2. We observed that the level of pro-inflammatory cytokines (TNF-α, IL-1β and IL-6) peaked at 4 days post-injury. In diabetic mice, we observed aberrant and significantly elevated levels of TNF-α, IL-1β and IL-6 compared with control and WP-treated diabetic mice from 4 to 13 days post-injury, which indicates a prolonged pro-inflammatory phase during the healing of diabetic wounds. By contrast, the level of IL-10 was significantly reduced in diabetic mice compared with control and WP-treated diabetic mice at the same time points. Thus, WP supplementation during diabetes significantly restored the levels of TNF-α, IL-1β, IL-6 and IL-10.

### The effects of WP supplementation on the expression of wound tissue chemokines and TGF-β

**Figure 3:**
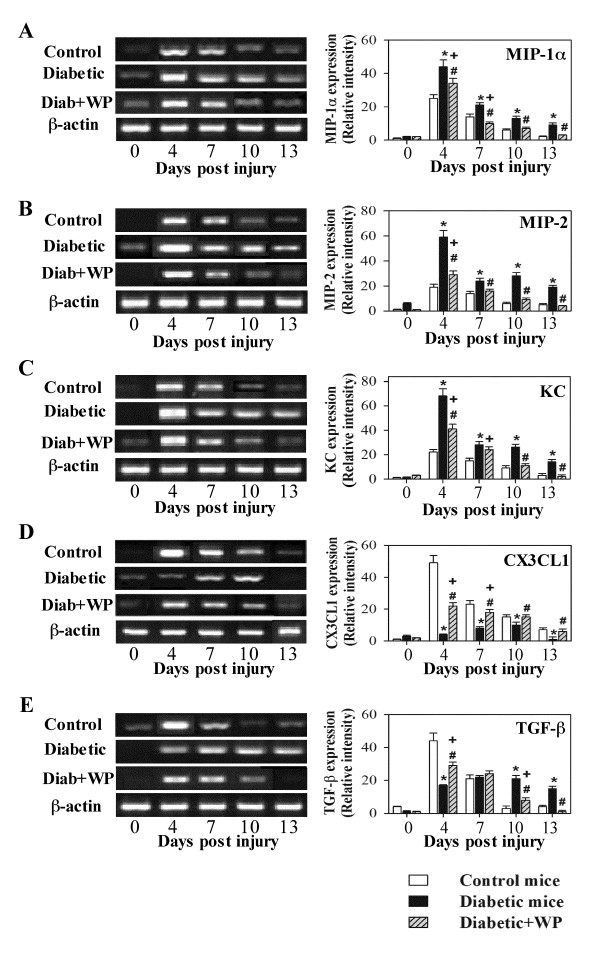
**Analysis of inflammatory chemokines, CX3CL1 and TGF-β1 in wound tissues.** Inflammatory chemokines, CX3CL1 and TGF-β1 were detected in non-wounded (Day 0) and wounded (4, 7, 10 and 13 days) skin of the same animals in the 3 groups of mice. (**A**-**E**) Representative RT-PCR results from three independent experiments with three animals per group are shown. (**B**) The ratios of RT-PCR signals for the indicated effectors to β-actin were calculated. Values represent the mean ± SEM. ^*^P < 0.05, diabetic vs. control; ^+^P < 0.05, diabetic + WP vs. control; ^#^P < 0.05, diabetic + WP vs. diabetic (ANOVA with Tukey’s post-test).

The expression levels of chemokines and growth factors, which play important roles in the process of wound healing, were measured by RT-PCR. Excisional wound tissues were collected from the 3 groups of mice on days 0, 4, 7, 10 and 13 post-injury. One representative experiment is shown for the expression of each MIP-1α, MIP-2, KC and TGF-β (i.e. one representative experiment for each gene expression (left page) and accumulated data in the bar graphs for each gene represented (right page) Figure 3. Day 0 represents one hour prior to wound induction (non-wounded skin tissue). The data from 10 individual mice from each group reveal that the levels of MIP-1α, MIP-2 and KC were significantly elevated for a prolonged period in the wound tissue of diabetic mice compared with control mice. WP-treated diabetic mice exhibited partially restored chemokine levels in wound tissue compared with control and diabetic mice (Figure 3 A, B, C). Similarly, the expression of TGF-β peaked at 4 days post-injury, and in WP-supplemented diabetic mice, TGF-β levels were significantly decreased, especially at days 10 and 13 post-injury, when compared with diabetic mice (Figure 3 E). By contrast, the levels of CX3CL1 were significantly reduced in diabetic mice when compared with control mice, and supplementation with WP partially restored these levels in diabetic mice (Figure 3 D).

## Discussion

Although it seems that the role of nutrition is well established in immune system functions and inflammatory diseases, little is known about the role of nutritional status in normal physiological processes, such as cutaneous wound healing [6]. Impaired wound healing in diabetic patients represents a severe complication of the disease and, more important, is an ongoing medical problem associated with significant mortality [31]. Therefore, several attempts have been made to understand the underlying defects in wound healing. In this study, we monitored the macroscopic changes and percentage of wound closure, which reflect the effects of wound contraction and healing. We observed that macroscopic changes and the rate of wound closure were significantly enhanced in diabetic mice supplemented with WP when compared with untreated diabetic mice. The accelerated closure of WP-treated diabetic wounds may be attributed to increased glutathione synthesis and cellular antioxidant defense [19]. Data obtained during the optimization of the parameters and conditions of the animal models during our investigation revealed that delayed wound repair in diabetic mice was associated with a significant increase in blood glucose levels and an obvious decrease in insulin levels, both of which were reversed by WP supplementation. Similarly, the addition of whey to meals has been observed to stimulate insulin release and reduce postprandial blood glucose excursion after a lunch meal consisting of mashed potatoes and meatballs in type 2 diabetic subjects [32]. Interestingly, we observed that WP treatment significantly decreased the elevated levels of pro-inflammatory cytokines (IL-1β, IL-6, and TNF-α) and increased IL-10 in plasma and wound tissue. Thus, WP limits prolonged inflammation, and these data elucidate the mechanism underlying the enhanced immune response and may be a cause for improving wound healing in WP-treated diabetic mice. These results are in agreement with those obtained by Peranteau et al., [10] who reported that overexpression of IL-10, an anti-inflammatory cytokine, decreased the inflammatory response to injury, creating an environment conducive for regenerative adult wound healing. In addition, a previous study that supports our results demonstrated that lactoferrin can regulate the levels of TNF-α and IL-6, which would decrease inflammation and mortality [33]. Several studies have focused on the critical roles of chemokines, such as MIP-1α, MIP2, and KC, during tissue repair processes [34,35]. In the present study, treatment of diabetic mice with WP may exert different effects that obviously increased the expression of MIP-1α, MIP2, and KC, and these effects may be participate in accelerating healing of diabetic wounds. Mori et al., [29] similarly demonstrated that several chemokines, such as MIP-1α and MIP2, have chemotactic activities toward neutrophils and macrophages and that their expression can be upregulated by other pro-inflammatory cytokines, such as IL-1β. TGF-β plays an important role in wound repair by adding signals important for the initiation of the healing cascade and by attracting macrophages and stimulating them to secrete additional cytokines, including fibroblast growth factor (FGF), platelet-derived growth factor (PDGF), TNF-α and IL-1 [36]. We also observed that WP treatment during diabetes decreased the expression of TGF-β in the wounded area and may be in turn promoting wound healing. Previous studies have demonstrated improvements in wound healing by altering growth factor and collagen expression [37]. CX3CL1 also contributes to wound healing by recruiting macrophages. In this study, impaired wound healing in diabetic mice was accompanied by a significant decrease in the levels of CX3CL1, which was partially restored by WP supplementation. WP-induced CX3CL1 enhances phagocytosis and the immune response; thus, WP may be a promising drug candidate for immunomodulation in chronic diabetic wounds. This study suggests that the oral administration of camel WP may be a new avenue for the treatment of skin wounds in diabetic patients.

## Conclusions

Taken together, the data presented in this study expand our knowledge of the benefits of WP supplementation in improving the healing and closure of diabetic wounds, suggesting that WP may be a promising drug candidate for treating diabetic wounds and their associated complications.

## Abbreviations

IL, Interleukin; KC, Keratinocyte-derived chemokine; MIP-1α, MIP-2 macrophage inflammatory proteins 1α and 2; STZ, Streptozotocin; TGF-β, Transforming growth factor-β; WPs, Whey proteins.

## Competing interests

The authors declare that they have competing interests.

## Authors’ contributions

GB put the design of the study, carried out the immunological assays, prepared figures, drafted the manuscript and performed the statistical analysis. BMB carried out some immunological parameters and participated in the analysis of the data. MHM was responsible for monitoring the food and water consumption for the animal model throughout the experiment period and participated in drafting the manuscript. MM was responsible for the animal model and participated in preparing the figures. OG participated in the statistical analysis and drafting the manuscript. All authors read and approved the final manuscript.

## Authors’ information

Dr Gamal Badr principle investigator of several research projects funded by King Saud University, as well as head of a research group of Immunology. Dr Badr is a leading and internationally respected immunologist. He has obtained his master and PhD in Immunology from Faculty of Medicine, Paris XI University, France with a scholarship from the Egyptian government as well as from Sidaction (France). He followed 2 years of significant postdoctoral experience at University of Montreal, Canada with fellowship from FRSQ. He awarded for the best oral presentation of Post-doctors in the 10th Annual Conference of CHUM (18 December 2007), Montreal University, Canada. He hold his associate professor appointment in Immunology at Assiut University, Egypt as well as at King Saud University (January 2011).
